# MDCT and MRI for the diagnosis of complex fractures of the tibial plateau: A case control study

**DOI:** 10.3892/etm.2013.1380

**Published:** 2013-11-01

**Authors:** YUNQIN XU, QIANG LI, PEIHUA SU, TUGANG SHEN, YAZHONG ZHU

**Affiliations:** Department of Orthopedic Surgery, 98th Hospital of PLA, Huzhou, Zhejiang 313000, P.R. China

**Keywords:** tibial fractures, X-ray computed tomography, magnetic resonance imaging, diagnosis

## Abstract

The aim of this study was to evaluate the clinical value of multidetector-row computed tomography (MDCT) and magnetic resonance imaging (MRI) in the diagnosis and treatment of complex fractures of the tibial plateau. A total of 71 patients with complex fractures of the tibial plateau (estimated Schatzker classifications III, V and VI) were included in this study. The X-ray, MDCT and MRI data obtained from the patients were analyzed. MDCT was the most sensitive method in the diagnosis of tibial articular surface collapse, cruciate ligament tibial avulsion fracture, degree of fracture comminution and degree of fracture displacement (P<0.01). MRI was the most sensitive method in the diagnosis of injuries of the cruciate and collateral ligaments, menisci and cartilage peeling of the articular surfaces (P<0.01). MDCT and MRI were demonstrated to be more sensitive than X-rays for the diagnosis of insidious damage around the knee.

## Introduction

Complex fractures of the tibial plateau commonly occur in patients following high-energy trauma, typically accompanied by severe damage to the knee articulation and the surrounding tissues. The diagnosis and treatment of complex tibial plateau fractures remains a significant challenge in orthopedic trauma (1,2). In the past, normal X-ray films were applied in the Schatzker classification of tibial plateau fractures; however, the information provided by the normal films is limited and does not satisfactorily assist clinical orthopedic physicians in the diagnosis and treatment process. In the present study, images from X-ray films, multidetector-row computed tomography (MDCT) and magnetic resonance imaging (MRI) were systematically analyzed and various indices, such as fracture location, displacement degree, degree of comminution and degree of joint surface collapse were analyzed. The ability to identify injuries to the menisci of the knee joint and cruciate and collateral ligaments, as well as insidious fractures, preoperatively to enable a precise diagnosis and the appropriate surgical treatment was evaluated. A total of 71 patients with complex fractures of the tibial plateau who were admitted to hospital from March 2004 to January 2009 were included in the study and their treatment and its effects are reported.

## Patients and methods

### Clinical data

Over a 5-year period, from March 2004 to January 2009, 92 patients with complex fractures of the tibial plateau were admitted to the 98th Hospital of PLA, Huzhou, China. Seventy-one of the patients (43 males and 28 females; mean age, 34.7 years) were systematically examined using X-ray, MDCT and MRI and underwent surgical treatment. A total of 40 patients presented with left tibial plateau fractures, while 31 patients presented with right tibial plateau fractures. According to the Schatzker classification (3), eight fractures were type III, 36 were type V and the remaining fractures were type IV. The causes of the fractures were as follows: 38 due to traffic accidents; 15 due to falls from high places; 10 due to injury by heavy objects and eight due to falls from bicycles or battery-powered bikes. There were 14 patients with potentially fatal injuries to the visceral organs and head, seven patients with other fractures around the knee joint, 10 patients with injuries to the anterior cruciate ligament, seven patients with injuries to the posterior cruciate ligament, eight patients with injuries to the collateral ligament, six patients with injuries to the menisci and three patients with injuries to the genicular vascular nerves. The patients were diagnosed and treated by the same group of physicians. Patients that it was not possible to examine by MRI and MDCT and those with injuries that were classified as Schatzker types I, II and IV were not included in the study. The study was approved by The Ethics Committee of The Medical Ethics Committee of The 98th Hospital of People’s Liberation Army (Huzhou, China). Informed consent was obtained from all included patients.

### Examination methods and diagnosis

#### X-ray or computed radiography (CR)

A 500 mA X-ray machine (Beijing Wandong Medical Equipment Co., Ltd., Beijing, China) was utilized in the X-ray examinations with a Lekai film, while an Agfa MD40 IP plate (Agfa Healthcare NV, Mortsel, Belgium) was selected for use in the CR examinations. The IP plate was scanned with a laser in the Agfa DC Solo system, prior to the scanned image being presented to the workstation to be handled. Following this, the film was printed using a Kodak 8900 laser printer (Kodak, Rochester, NY, USA). The anterior and lateral sides of the injured knee joint were imaged.

#### Computed tomography (CT) scanning and 3D reconstruction

The patients’ injured knee joints were scanned by a Siemens Symphony 16-row spiral CT scanner (Siemens AG, Erlangen, Germany). The scanning slices were 5 mm deep, with slice intervals of 3 mm. A 0.75 mm bone algorithm was adopted subsequent to scanning, and multiplanar reconstruction (MPR) and observations of the axial, sagittal and coronal positions were performed. The reconstruction slices were 2–3 mm thick. The MPR images were adjusted and analyzed by two highly qualified radiologists and two highly qualified orthopedists subsequent to the images being handled in the workstation. Multidirectional adjustment of the MPR images was performed to show the cross section and the sagittal, coronal and inclined planes (the inclined plane image was based on the plane in which there was a 30º and 60º angle with the cross section and the sagittal and coronal planes) of the target bones, according to the bone structure. An any-angle inclined plane image adjustment and curve imaging were applied to show the fractures if necessary. The trend of the fracture line was tracked to define the area affected by the fracture.

#### MRI examination

The patients’ injured knee joints were examined using a Signa 1.5 T HD scanner (GE Healthcare, Waukesha, WI, USA), while the patients lay straight and in a supine position. The feet entered the scanner first and then the knees were turned outward by 10–15º. Rapid spinning echo and spectrum pre-saturated inverse recovery sequences were used for the sagittal, coronal and axial scanning. The scanning parameters were as follows: T1WI TR 500 msec, TE 17 msec; T2W1 TR 4,000 msec, TE 100 msec; BWI/SPIR sequence: TR 3,000 msec, TE 10 msec; 4-mm-thick slices; slice interval, 0.4 mm; scope, 150 ram; matrix, 256×512; stimulating times, twice.

The appropriate treatment was determined according to the patient’s condition and degree of limb turgidity, the radioactive assessments (X-ray film, CT and MRI), the skill of the surgeon and the patient’s medical history. Patients were treated with plaster immobilization or calcaneus traction, with ice bags for external application. The injured limb was elevated and dehydrated, dexamethasone sodium phosphate was applied and any bleeding was stopped. Potentially fatal trauma was treated first, prior to the patient being treated with open reduction and anatomical reduction when the condition of the patient was stable and turgidity was apparent (5–14 days).

### Double incision and single plate

This group comprised 24 patients in total (14 males and 10 females; mean age, 33.6 years) and the average follow-up period was 31.4 months. The incision was initiated at the superior edge of the lateral tibia and extended to beneath the tibial nodule. The length of the incision was ~15 cm. The skin, subcutaneous tissues, deep fascia and the attachment point of the muscle group were cut open successively, in order to expose the lateral tibial platform. Following this, the coronal ligament was cut open and the tibial articular surface was exposed. The tibial medial plateau was reduced using Kirschner wire with the assistance of C-shaped arm X-ray fluoroscopy. The L-shaped supporting plate, golf plate or locking plate was subsequently placed and fixed on the lateral tibia. Bone from the patient’s ilium or a bone allograft was implanted if necessary. There were three patients whose genicular medial collateral ligament was reduced using a genicular medial incision.

### Double incision and bilateral plates

This group comprised 21 patients in total (12 males and nine females; mean age, 33.2 years) and the average follow-up period was 28.4 months. On the basis of the genicular anterior incision, two other incision were made in the posterior edge of the medial plateau of the tibia, which were 12–15 cm long and >8 cm wide. The skin, subcutaneous tissues and deep fascia were cut open successively to the posterior edge of the medial tibial plateau. Following this, the tibial medial plateau and the posterior edge beneath the periosteum were peeled and exposed. Based on the preoperative CR films, CT, MRI and degree of joint surface collapse, the medial plateau was reset first, prior to the medial plateau being taken as the mark to reset the lateral plateau fracture and to fix the double plates with screws. Bone from the patient’s ilium or a bone allograft was implanted according to the condition of the patient. There were two patients whose collateral ligaments were sewn up and restored, and one patient whose menisci were excised.

### Bilateral plates via genicular anterior midline incision

This group comprised 26 patients in total (17 males and nine females; mean age, 35.3 years) and the average follow-up period was 23.6 months. The genicular anterior midline incision was 18–20 cm long and was initiated from a point 2 cm above the scutum, prior to being extended to the central and upper crest of the tibial diaphysis and then through the middle scutum, to terminate at the tibial nodule. The skin, subcutaneous tissues and deep fascia were cut open successively until the condyles were exposed beneath the deep fascia. The surgical method was designed according to the preoperative X-ray film, CT and MRI of the knee joint. The genicular medial joint capsule was only cut open if the patient’s medial joint surface had collapsed. The periosteum was peeled to expose the broken side of the lateral tibial plateau. Then, after conducting temporary fixation with Kirschner wire, the reconstruction plates were fixed in place with screws, and the muscle group in front of the tibia was then cut open with an electric knife at the point of the lateral tibial plateau. The broken side of the lateral tibial plateau was peeled and exposed simultaneously. The meniscal coronal ligament was cut open and the joint surface was reset in direct view. With regard to posterior joint surface collapse, a tibial plateau bone knife was used for vertical splitting, in order to expose the collapse site of the joint surface. The surface was then impacted with a periosteal peeling tool until the joint surface became flat, and bone from the patient’s ilium or a bone allograft was implanted in the empty sites. L-shaped supporting plates were placed and fixed with screws once the fracture had been reset. There were 15 patients whose cruciate ligaments were repaired with steel or absorbed wire. Three patients had posterior cruciate ligament tibial avulsion fractures and collapse fractures of the posterior tibial plateau, which were repaired through an assistant incision behind the knee. There were three patients whose collateral ligaments was sewn and repaired and three patients whose menisci were excised. In addition, there were two patients whose popliteal arteriovenous vessels were examined and repaired using assistant incision behind the knee. One patient’s peroneal nerve was examined and decompressed.

### Indices and methods

Each patient’s X-ray, MDCT and MRI examination images were examined and a quantitative statistical satisfaction score, showing indices such as fracture location, degree of fracture comminution, degree of fracture displacement and degree of bone defect, was assigned. The standard of evaluation was based on the imaging information, with scores as follows: 0 if the fracture was not visible, 1 if the fracture was not clearly visible and 2 if the fracture was clearly visible; 0 if the degree of fracture comminution was not visible, 1 if the degree of fracture comminution was visible but vague and 2 if the degree of fracture comminution was clear; 0 if the degree of fracture displacement was not visible, 1 if the degree of fracture displacement was visible but unclear and 2 if the degree of fracture displacement was clearly visible; 0 if the bone defect was not visible, 1 if the bone defect was visible but unclear and 2 if the bone defect was clearly visible. An assessment of 2 points was considered ‘satisfactory’, 1 point was considered ‘moderately satisfactory’ and 0 points was considered ‘unsatisfactory’. Statistical scores were assigned for collapsed joint surfaces and injuries of the cruciate ligament, menisci and collateral ligament using X-ray, MDCT and MRI examinations, respectively.

### Statistical methods

The data were processed and analyzed using SPSS statistical software version 11.5 (SPSS, Inc., Chicago, IL, USA). A metering method was used to evaluate fracture position and number of cases, while single factor and 3-level variance analysis were used when comparing the scores of the three groups. A Q-test (Newman-Keuls method) was used when comparing two mean numbers and a χ^2^ test was to compare every two of several sample rates. P<0.05 was considered to indicate a statistically significant difference.

## Results

A total of 71 patients underwent X-ray (or CR), MDCT and MRI examinations of injured knee joints in the present study. The average follow-up period was 31.2 months, the average duration of surgery was 1.9±0.4 h and the average bleeding volume was 446.5±107.9 ml. The infrapatellar branch of the saphenous nerve was injured in one patient during the surgery and healing by first intention of fracture was performed in 71 patients. One patient recovered following a postponement. In the surgical treatment, bone from the patient’s ilium was excised and fixed within 8 months subsequent to the injury. The injury then typically recovered without further complications within the following 10 months. Two patients suffered from superficial and deep infections following the surgery, one of which was skin ischemic necrosis combined with soft tissue defects, bone and plate exposure and superficial infection. This was treated by debriding the wound and covering the retrograde island flap pedicled with the saphenous nerve nutrient vessel. The other patient suffered from a deep infection of the wound and acute pyogenic osteomyelitis. Following treatment with a pure drip for 4 weeks, which demonstrated no efficacy, the internal fixation was removed and an external fixation bracket was used for the fixation instead. This, in combination with the drip, cured the patient.

**Figure 1:**
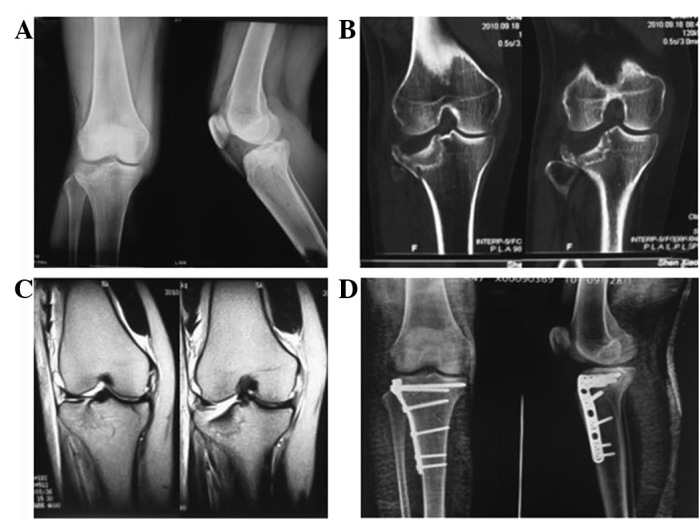
Female, 38 years old, with collapse fractures of the right tibial platform. (A) Preoperative X-ray. (B) Preoperative computed tomography (CT) scan showing the lateral central collapse of the right tibial platform of ~2 cm. (C) Preoperative magnetic resonance imaging (MRI) scan showing the lateral tibial plateau fracture and the complete disruption of the medial collateral ligment. (D) Postoperative X-ray images showing the anatomical reduction of the right lateral tibial plateau fracture and the flat joint surface.

**Figure 2:**
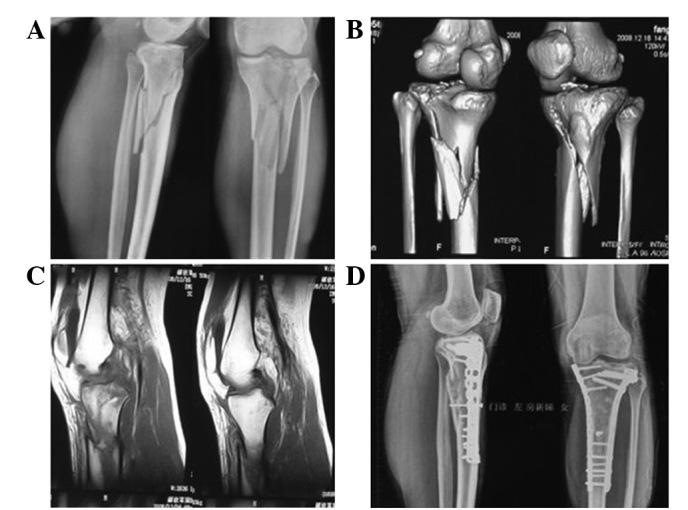
Female, 38 years old, with a serious fracture of the left tibial plateau. (A) Preoperative X-ray. (B) Preoperative computed tomography (CT) and 3D reconstruction showing the serious fracture of the left tibial plateau, with obvious joint surface collapse and fractures of the anterior cruciate ligament. (C) Preoperative magnetic resonance imaging (MRI) showing the fracture of the left tibial plateau. The anterior cruciate ligament was abnormal. (D) One year subsequent to surgery, the X-ray showed that the left tibial plateau fracture fixation was good, and that the joint surface was flat.

**Figure 3:**
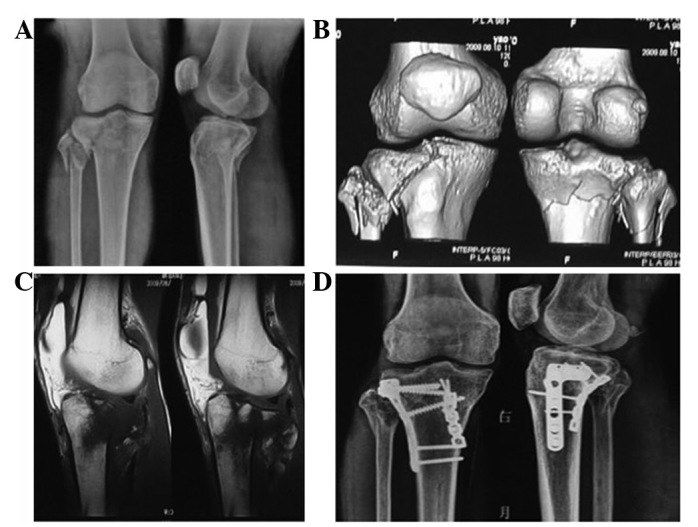
Male, 39 years old, with a tibial plateau fracture of the right tibial platform and a comminuted fracture of the small head of the fibula. (A) Preoperative X-ray. (B) Preoperative computed tomography (CT) and 3D reconstruction showing the comminuted fracture of the right tibial plateau, the collapse site in the lateral and posterior plateau and the comminuted fracture of the small head of the fibula. (C) Preoperative magnetic resonance imaging (MRI) showing the comminuted fracture of the right tibial plateau. The interior and posterior plateau collapses were serious. (D) One year following surgery, the X-ray showed that the right tibial plateau fracture fixation was good and that the joint surface was flat.

The conditions of the complex fractures of the tibial plateau, as demonstrated by the three types of imaging examination, are shown in Tables I and II. MDCT was the most sensitive method in the diagnosis of tibial articular surface collapse, cruciate ligament tibial avulsion fracture, degree of fracture comminution and degree of fracture displacement. MRI was the most sensitive method in the diagnosis of injuries of the cruciate and collateral ligaments and the meniscus and cartilage peeling of the articular surface. Typical cases are illustrated in Figs. 1–3.

## Discussion

Certain types of fractures may be identified solely by X-ray examination; however, fractures of the tibial plateau, particularly complex fractures of the tibial plateau, require evaluation by CT and MRI (4–7). MDCT is efficacious in the classification of fractures of the tibial plateau and enables observations of the morphology and the degree of displacement from different levels and angles. In particular, CT 3D reconstruction forms an intuitive image through the rotation and incision of the axis, which enhances the recognition of anatomical structures and pathological changes by displaying the images from different spatial angles. This avoids the blind zone caused by vision limitations, thus improving the diagnostic level and enabling evaluations of the degree of displacement of the fractures to be performed in a variety of planes. X-ray films solely show data from the anterior and lateral planes, which do not provide a satisfactory pathological anatomical image for the clinical surgeons. With regard to the diagnosis of fracture location, degree of fracture comminution, joint surface collapse and fracture displacement, the use of MDCT was superior to that of X-ray and MRI; however, the use of MRI was conducive to diagnosing insidious fractures and bone contusions that were not revealed by X-ray or CT, particularly with regard to damage of cartilage inside the joint and soft structures. MRI was also able to identify injuries of the menisci, the anterior and posterior cruciate ligaments and the medial and lateral collateral ligament, demonstrating incomparable advantages over X-ray and CT. In the present study, six patients were diagnosed with injuries to the menisci using preoperative MRI: 17 were diagnosed with cruciate ligament injuries, one was diagnosed with cartilage peeling from the joint surface of the femoral condyle and 18 were diagnosed with an insidious fracture of the femoral condyle or bone contusions. The study demonstrated that MDCT was able to show the condition of the tibial plateau fractures precisely, while MRI was beneficial in the early detection of damage to the soft structures inside the knee joint, such as injuries of the menisci and the cruciate and collateral ligaments and the degree of the injuries (8).

It is widely recognized that the majority of the surgical regimens planned for the treatment of tibial plateau fractures that have been based on common X-ray films require some improvement or adjustment following CT and MRI evaluations. MDCT and MRI are able to be more precise in showing the classification of fractures and improving the surgical plan than X-rays (6). A precise and reasonable judgment may be provided using X-ray, MDCT and 3D reconstruction and MRI, which enables clinical surgeons to design an optimal surgical plan according to the pathological morphologies and anatomical characteristics. The size of a fracture fragment may be precisely measured using imaging information, enabling a pre-reduction to be conducted in advance using spatial imagination; furthermore, an appropriate inner fixation object of the correct size and a placement site may be selected, and the placement and direction of the screws for fixation may be determined using imaging data. An appropriate incision for exploration may be selected prior to surgery, combined with the pathology requiring surgical repair inside the articular cavity, such as injuries of the menisci, ruptures of the cruciate ligaments, avulsed fractures and ruptures of the medial and lateral collateral ligaments. If genicular cavity exploration is required, a genicular anterior midline incision is selected. The incision location may be adjusted either laterally or medially, according to the requirement of the procedure. It is beneficial to the accomplishment of the genicular cavity exploration and surgical treatment if the incision extends towards the appropriate site in the body. If it is not necessary to explore the pathology of the articular cavity and only restoration of the fat percentage of the joint surface is required, genicular medial and lateral double incision or a genicular anterolateral posteromedial incision is selected to reduce the surgical trauma and to expose solely the tissues requiring surgical treatment. This leads to a desirable treatment efficacy. In the present study, there were no injuries of the knee joint that were not diagnosed, no prolonged surgeries, no unnecessary trauma and no bleeding during surgery. The infection rate of the wounds was low and good functional recovery of the knee joints following surgery was observed.

It has been demonstrated that MRI and MDCT may be used to assist in the identification of insidious fractures of the distal tibia that are involved with the joint surface (9). For patients with fractures of the tibial plateau, the drawer and inside and outside turning tests may not always be conducted satisfactorily, due to the early swelling and pain of the limbs. It is not possible to identify injuries of the cruciate and collateral ligaments and the menisci by common X-ray examination. However, early damage of the genicular cavity may lead to a leakage outside the joint due to arthroscopic examination, thus preventing a further arthroscopic examination of the knee joint from being conducted (10). If an MRI examination is not conducted in time, it may delay the diagnosis or lead to missed diagnoses, resulting in unnecessary medical disputes. MDCT and MRI enable fractures and bone contusions around the knee joint and injuries of the menisci and ligaments to be diagnosed as soon as possible. Furthermore, MRI reveals bone contusions and the insidious fractures that are not able to be diagnosed using common X-ray films. The existence of bone contusions indicates that there are injuries of the joint cavity and ligament, requiring evaluation by MRI (11). Insidious fractures may be identified as promptly as possible using MDCT scanning. The efficacy of the treatment may be improved by the design of a more appropriate surgical plan. As a result, MDCT and MRI have potential as relatively new imaging technologies that may be applied to complex fractures of the tibial plateau.

## Figures and Tables

**Table I:** Comparison of the satisfaction scores from X-ray, MDCT and MRI for the diagnosis of fractures of the tibial plateau.

Variable	Case (n)	Fracture sites	Severity of bone comminution	Fracture displacement	Bone defects
X-ray	71	1.04±0.20	0.81±0.51	1.23±0.48	0.36±0.51
MDCT	71	1.82±0.38^a^	1.92±0.26^a^	1.92±0.26^a^	1.55±0.79^a^
MRI	71	1.12±0.33^b^	0.83±0.60^b^	0.46±0.58^a^^b^	0.26±0.53^b^
F	-	131.06	119.99	173.05	91.46
P-value	-	<0.01	<0.01	<0.01	<0.01

Scores are presented as the mean ± standard deviation.

a P<0.01 compared with X-ray film;

b P<0.01 compared with multidetector-row computed tomography (MDCT).

MRI, magnetic resonance imaging.

**Table II:** Comparison of examination results from X-ray, MDCT and MRI for the diagnosis of knee injuries.

Variable	Case (n)	Joint surface collapse	Other (cruciate ligament+meniscus+collateral ligament injury)
X-ray	71	16	4(3+0+1)
MDCT	71	61^a^	11^a^(10+0+1)
MRI	71	12^b^	31^a^^b^(17+6+8)
χ^2^	-	85.7327	32.6626
P-value	-	<0.01	<0.01

a P<0.01 compared with X-ray;

b P<0.01 compared with multidetector-row computed tomography (MDCT).

MRI, magnetic resonance imaging.
